# Supplementary material for: Histone H2B ubiquitylation represses gametogenesis by opposing RSC-dependent chromatin remodeling at the ste11 master regulator locus
Source: eLife. 2016 May 12;5:e13500. doi: 10.7554/eLife.13500 (PMC4865366; doi:10.7554/eLife.13500)
Supplement: Supplementary file 1. — DOI: http://dx.doi.org/10.7554/eLife.13500.014 [file elife-13500-supp1.docx]

**Oligonucleotides used in this study**

*ste11* locus:

Amplicon **A**: located 2211 bp upstream of the *ste11* ATG

1017: gttggttgccaatgcgtatg

1018: tacgacgcgaaacaattcaacg

Amplicon **B**: located 121 bp downstream of the *ste11* ATG

968: tcgtcgagatcgccaagc

969: ggcactttcatttctccacagc

Amplicon **C**: located 1377 bp upstream of the *ste11* ATG

1015: ctttgtctctaccaccatagttatcc

1016: ccaagcgatagaataacgatgcg

Note that this amplicon was also used for the quantification of the mRNA in Q-RT-PCR

Amplicon **D**: located 1114 bp downstream of the *ste11* ATG

970: gccatacttcttacccagcaatg

971: cagaattggtaacactagacgaagag

*cdc14* locus:

1371: actgcatgtgattgaaggattgg

1372: tagatggctgagatggctg

Amplicon used for the quantification of the mRNA in Q-RT-PCR, located 338 bp downstream of the *cdc14* ATG:

*act1* locus:

Amplicon used for the normalization of the Q-RT-PCR, located 948 bp downstream of the *act1* ATG:

739: ccactatgtatcccggtattgc

740: caatcttgaccttcatggagct

Oligonucleotides used for nucleosome scanning (**Figure 3B**)

1F cgtttcccattttatgatcttgct

1R gacatgtaagatggagaacaagaatg

2F acttacctttccgacattcttgt

2R tcccttccatcatccctgg

3F tatttgtggtgcatgccatc

3R agtcattgccattggaattttgc

4F cgcctaattttcggtaaattgtagc

4R aaaaatacaggtactacgagcgg

5F gtgtggtttctttccttccg

5R gtagtttggggtgaacggaac

6F ccattcttatttcactcccgttc

6R gcatagcgaatgtgtgaaaaca

7F aactgccggcacttgttttc

7R cggcaatttgcgaaaccg

8F gcttaaaccttacctataataccctgc

8R tggagagttaaggaggggtg

9F tgctttctccacccctcc

9R gcaaggcaaaatgacaagaagc

10F cattggaagactagtcgttggc

10R gattagtgtggcacttggca

11F acgaaaagtagctttgcgagac

11R cttgcctacaaacgatgtaacaca

12F tccctgactttgtttgctgtg

12R ggggaagggacaaaattcaca

13F ctgtgaattttgtcccttcccc

13R ggcgtaatacgcgatgttactg

14F attgtcagtaacatcgcgtattacg

14R ctcactatgtaagtcaccaggtatgt

15F acatacctggtgacttacatagtgag (the amplicon #15 covers the +1 site)

15R caggtttaagattgaaaagtcagaatgc

16F acaaagcattattagcattctgacttttc

16R cgaggcaaaagctctcaaagaa

17F gcattttatttttctttgagagcttttgc

17R gctgcaatcaagacaaagacc

**Strains used in this study**

#581 *h+ lsk1::ura4 ura4-D18*

#552 *h- rpb1 S2A-kanR*

#701 *h-* *rpb1 S7A-kanR*

#1440 *h-* *pmk1::kanR*

#663 *h- ubp8::ura4 ura4-D18*

#913 *h-* *rsc1::kanR*

#1425 *h-* *arp9::kanR*

#1327 *h-* *snf22::kanR*

#782 *h-* *ubp16::kanR*

#783 *h-* *ubp8::ura4 ubp16::kanR ura4-D18*

#776 *h- htb1 SA-ST-KR-kanR*

*#*774 *h- htb1 SA-ST-kanR*

#640 *h- htb1 KR-kanR*

#662 *h- htb1 KR-kanR lsk1::ura4 ura4-D18*

#824 *h- ubp8::ura4 lsk1::kanR ura4-D18*

#1424 *h- hos2::hphR*

#1181 *h- h3.1/h4.1::kanR h3.2 K14R h3.3/h4.3::natR*

#1046 *h-* *rsc4::kanR*

#1145 *h- snf5::kanR*

*#*1183 *h- snf21-TAP-natR*

*#*1184 *h- snf21-TAP-natR ura4::rTetR-tup11*

#1191 *h- kanR-tetO-snf21-Tap-natR ura4::rTetR-tup11*

*#*1105 *h+ mst2::ura4 mst1::kanR leu1-mst1 L344S leu1-32 ura4-D18 ade6-M210* (a gift of S. Forsburg)

*#*1455 *h- h3.1/h4.1::kanR h4.2 K16R h3.3/h4.3::natR*

#1248 *h- arp9-TAP-natR*

#1591 *h- arp9-TAP-natR htb1 KR-kanR*

#1356 *h- arp9-TAP-natR hos2::hphR*

#1250 *h- arp9-TAP-natR lsk1::ura4 ura4-D18*

*#*911 *h-* *cdc14-118* (a gift of V. Simanis)

*#*642 *h-* *rhp6::kanR*

*#*1042 *h-* *rsc1-TAP-hphR*

*#*994 *h- lsk1::kanR rsc1-TAP-hphR*

*#1434 h- snf22-TAP-kanR*

*#1358 h^90^ snf22::kanR*

*#1055 h^90^ rsc1::kanR*

*#569 h^90^ rpb1 S2A-kanR*

*#568 h^90^*

*#568 h+ htb1 KR-natR lsk1-TAP-kanR*

*#772 h+ lsk1-TAP-kanR*
